# Supplementary material for: Radiation Synthesis of Selenium Nanoparticles Capped with β-Glucan and Its Immunostimulant Activity in Cytoxan-Induced Immunosuppressed Mice
Source: Nanomaterials (Basel). 2021 Sep 18;11(9):2439. doi: 10.3390/nano11092439 (PMC8469400; doi:10.3390/nano11092439)
Supplement: Supplementary file 1 [file nanomaterials-11-02439-s001.zip › nanomaterials-1365735-supplementary.pdf]

nanomaterials

Supplementary Materials

Radiation Synthesis of Selenium Nanoparticles Capped with β-Glucan and Its Immunostimulant Activity in Cytoxan-Induced Immunosuppressed Mice

Nguyen Thi Dung [1,2], Tran Duc Trong [1], Nguyen Thanh Vu [1], Nguyen Trong Binh [1], Tran Thi Le Minh [2] and Le Quang Luan [1,*]

[1] Biotechnology Center of Ho Chi Minh City, Ho Chi Minh City 700000, Vietnam; thuydung9810@gmail.com (N.T.D.); trong21052011@gmail.com (T.D.T.); ntvu1412@gmail.com (N.T.V.); nguyentrongbinhcnsh@yahoo.com (N.T.B.)

[2] Faculty of Biologinal Sciences, Nong Lam University, Ho Chi Minh City 700000, Vietnam; ttlminh@hcmuaf.edu.vn

* Correspondence: lequangluan@gmail.com

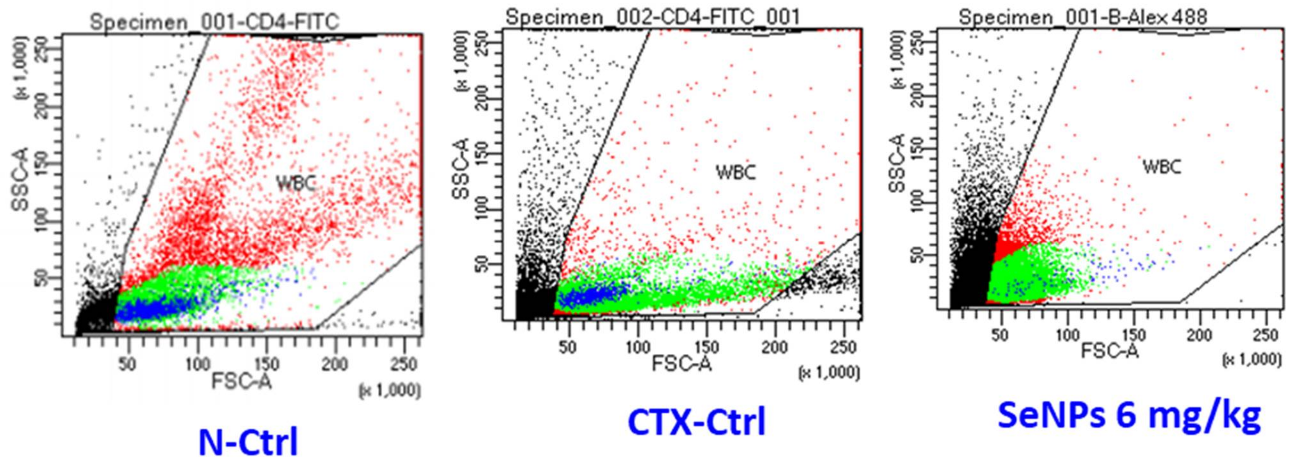

Figure S1. The typical flow cytometry scatter plots of WBCs in peripheral bloods of normal control mice received only distilled water (N-Ctrl), CTX-induced immunosuppressive mice received only distilled water (CTX-Ctrl) and mice supplied with 6 mg SeNPs kg^{-1} for 14 days.

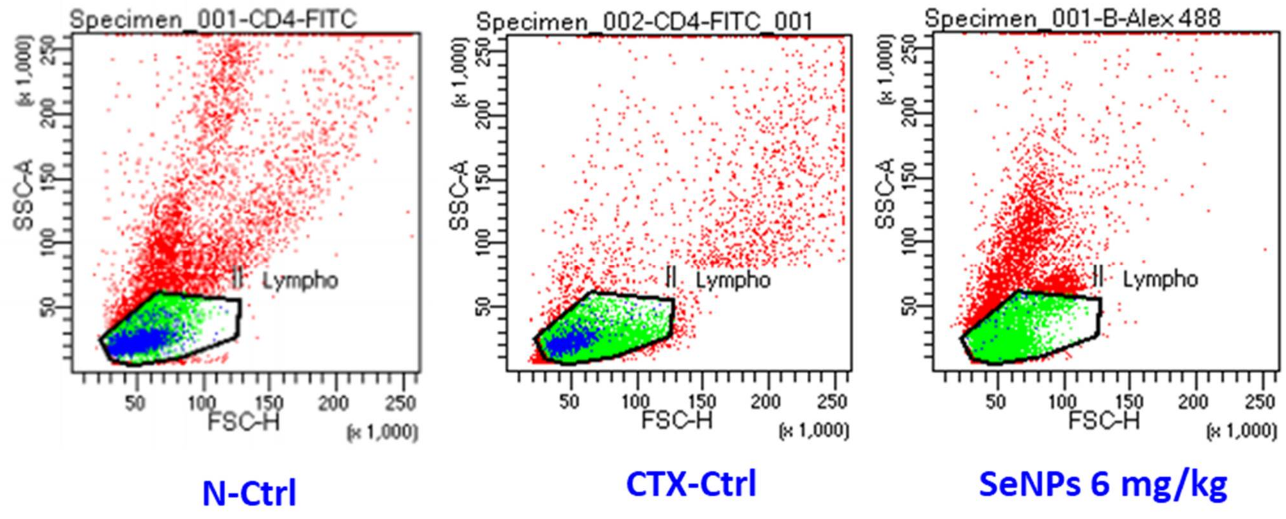

Figure S2. The typical flow cytometry scatter plots of lymphocyte in peripheral bloods of normal control mice received only distilled water (N-Ctrl), CTX-induced immunosuppressive mice received only distilled water (CTX-Ctrl) and mice supplied with 6 mg SeNPs kg^{-1} for 14 days.

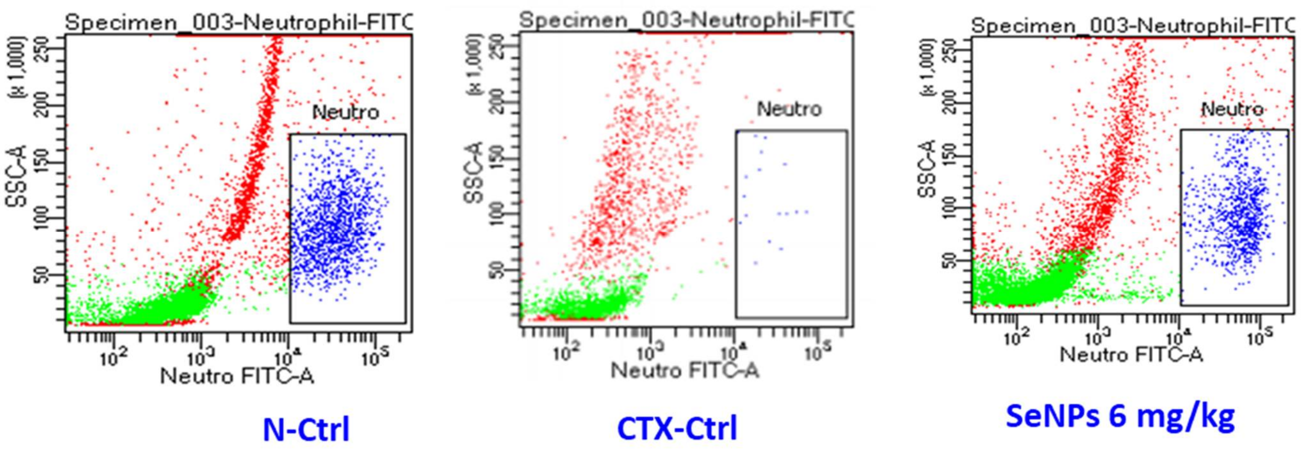

Figure S3. The typical flow cytometry scatter plots of neutrophil in peripheral bloods of normal control mice received only distilled water (N-Ctrl), CTX-induced immunosuppressive mice received only distilled water (CTX-Ctrl) and mice supplied with 6 mg SeNPs kg^{-1} for 14 days.

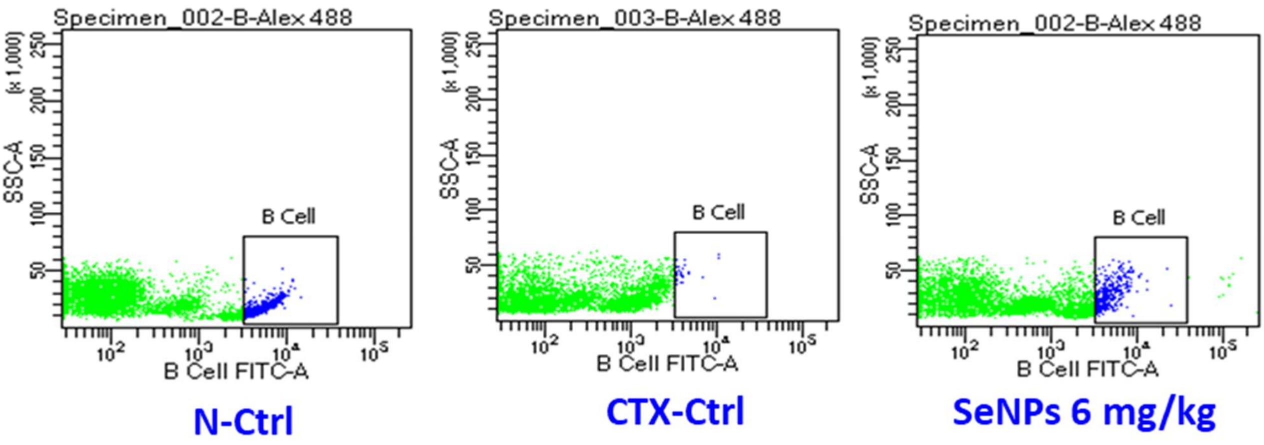

B cells in blood

Figure S4. The typical flow cytometry scatter plots of B cells in peripheral bloods of normal control mice received only distilled water (N-Ctrl), CTX-induced immunosuppressive mice received only distilled water (CTX-Ctrl) and mice supplied with 6 mg SeNPs kg^{-1} for 14 days.

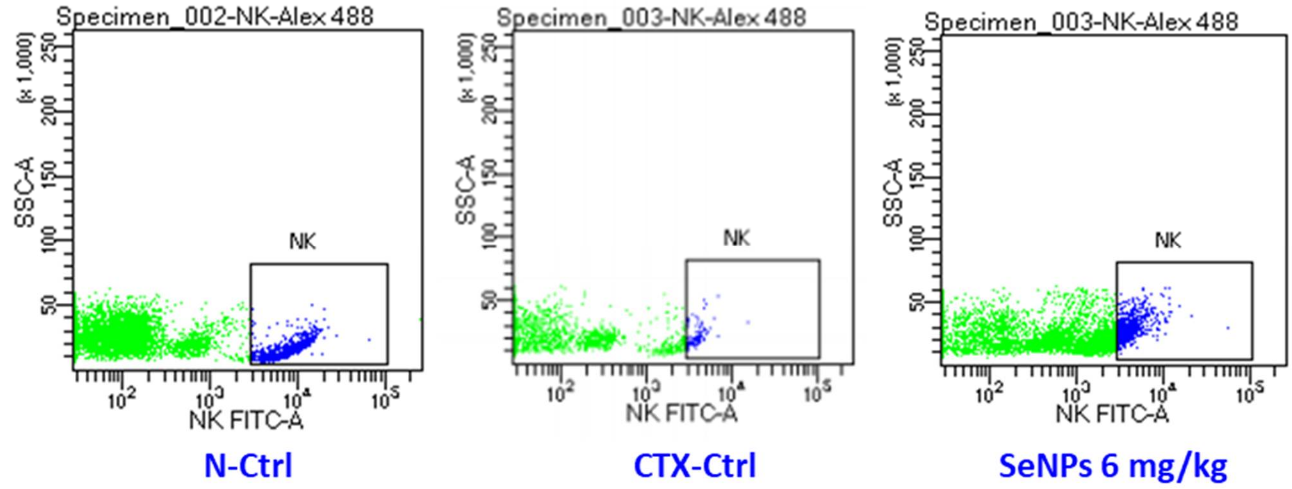

Figure S5. The typical flow cytometry scatter plots of natural killer cells in peripheral bloods of normal control mice received only distilled water (N-Ctrl), CTX-induced immunosuppressive mice received only distilled water (CTX-Ctrl) and mice supplied with 6 mg SeNPs kg^{-1} for 14 days.

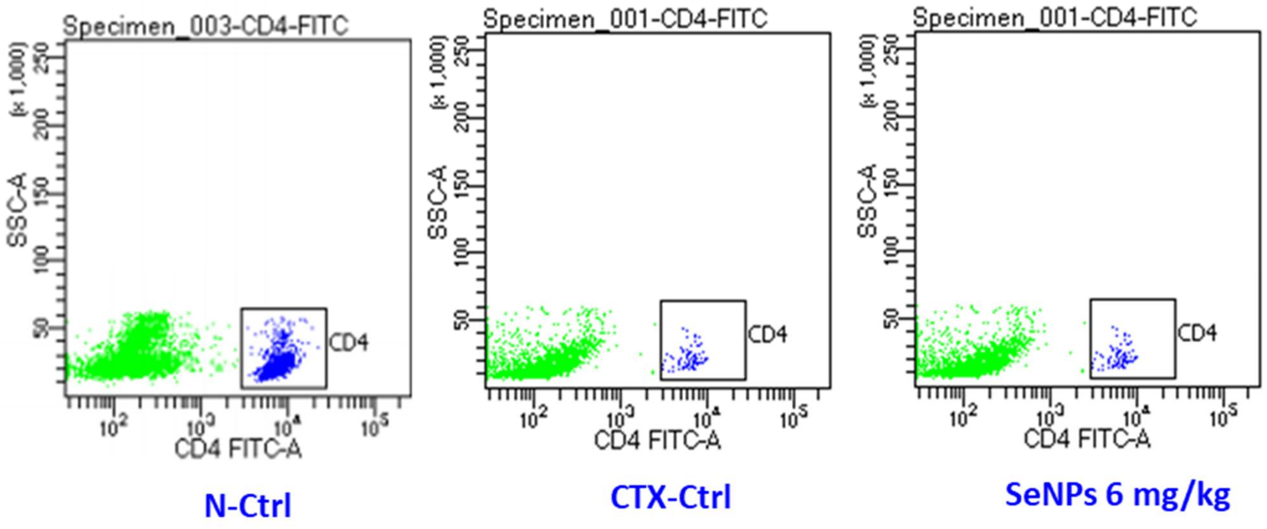

Figure S6. The typical flow cytometry scatter plots of CD4+ cells in peripheral bloods of normal control mice received only distilled water (N-Ctrl), CTX-induced immunosuppressive mice received only distilled water (CTX-Ctrl) and mice supplied with 6 mg SeNPs kg^{-1} for 14 days.

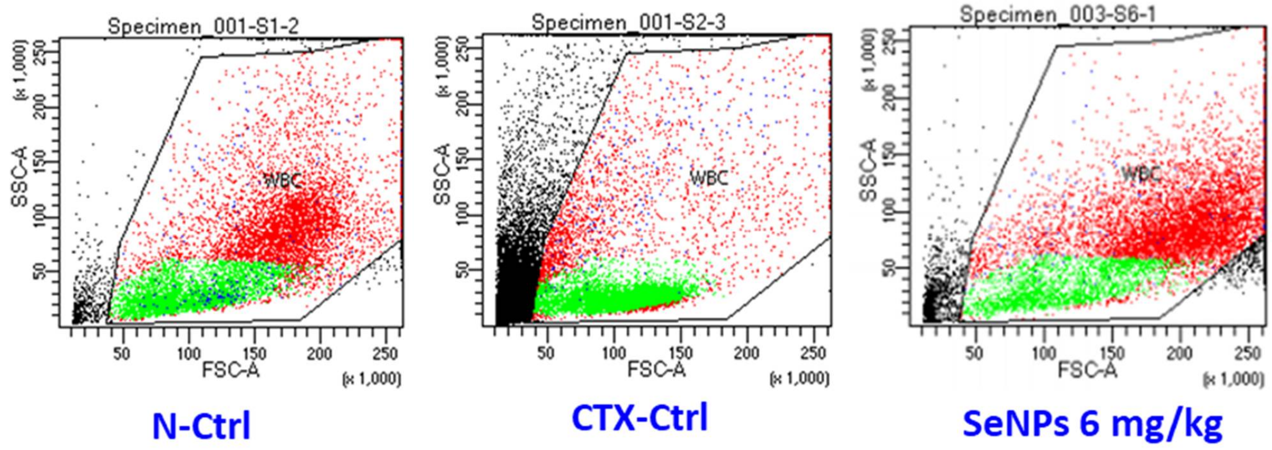

Figure S7. The typical flow cytometry scatter plots of WBCs in bone marrow of normal control mice received only distilled water (N-Ctrl), CTX-induced immunosuppressive mice received only distilled water (CTX-Ctrl) and mice supplied with 6 mg SeNPs kg^{-1} for 14 days.

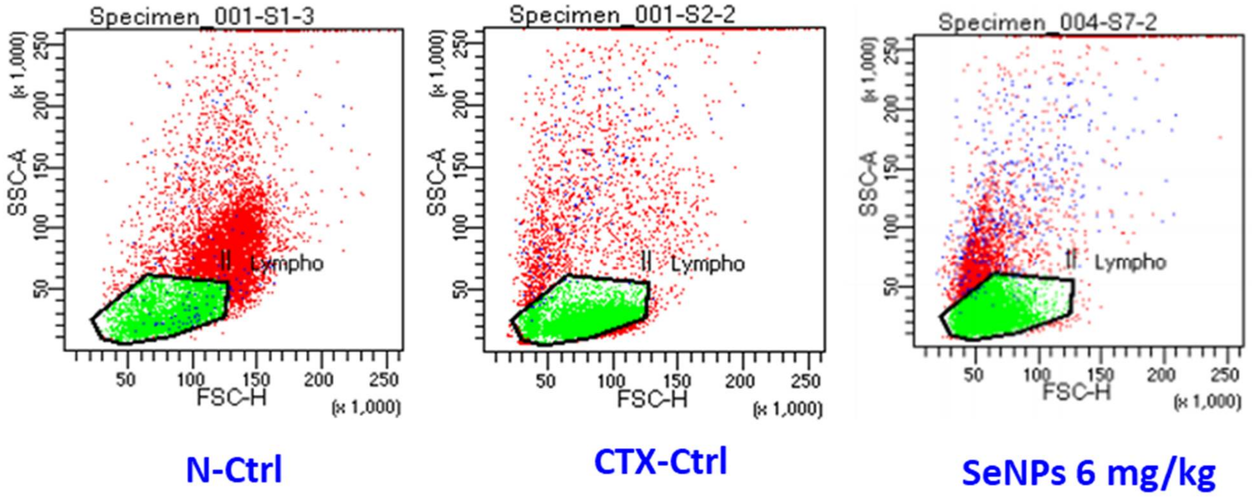

Figure S8. The typical flow cytometry scatter plots of lymphocytes in bone marrow of normal control mice received only distilled water (N-Ctrl), CTX-induced immunosuppressive mice received only distilled water (CTX-Ctrl) and mice supplied with 6 mg SeNPs kg^{-1} for 14 days.

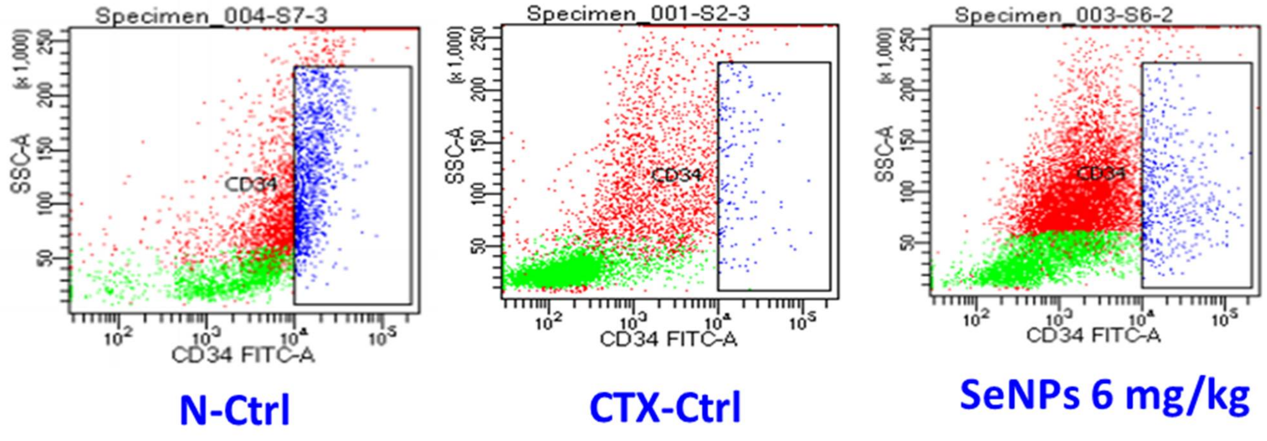

Figure S9. The typical flow cytometry scatter plots of CD34+ cells in bone marrow of normal control mice received only distilled water (N-Ctrl), CTX-induced immunosuppressive mice received only distilled water (CTX-Ctrl) and mice supplied with 6 mg SeNPs kg^{-1} for 14 days.
